# Evaluating the Influence of Different Recommended Dietary Levels of Cu and Zn on Finishing Pigs

**DOI:** 10.3389/fvets.2021.770195

**Published:** 2022-01-17

**Authors:** Meijun Li, Wei Tang, Peng Liao, Yunhu Li

**Affiliations:** ^1^Department of Animal Science, College of Animal Science and Technology, Hunan Biological and Electromechanical Polytechnic, Changsha, China; ^2^Hunan Tianxin Seed Industry Co., Ltd, Chenzhou, China; ^3^Institute of Subtropical Agriculture, Chinese Academy of Sciences, Changsha, China

**Keywords:** copper and zinc sources, digestive enzyme, fecal mineral excretion, finishing pig, recommended levels performance, digestive function

## Abstract

This study was conducted to evaluate the effects of dietary supplementation of different recommended levels of Cu and Zn on antioxidant capacity, tissue mineral status, minerals excretion, meat quality, digestive enzyme activity, and metal transporters in finishing pigs. A total of 120 pigs (with an average initial body weight (BW) of 70.0 ± 2.1 kg) were randomly divided into four treatments: (1) basal diet without added Cu or Zn (control), (2) basal diet+35 mg cupreous N-carbamylglutamate chelate (NCG-Cu) +150 mg zinc-methionine chelate (Zn-Met) (AC), (3) basal diet + 3.0 mg of NCG-Cu + 43 mg Zn-Met (CN), and (4) basal diet + 3.5 mg NCG-Cu + 50 mg Zn-Met (NRC100). Pig growth performance was not affected by the level of Cu or Zn. Among the four treatments, the AC treatment had the highest concentration (*P* < 0.05) of glutathione peroxidase (GSH-P*x*). Pigs fed the AC diet had the highest (*P* < 0.05) liver Zn, fecal Cu, and fecal Zn among the four treatments. The protein levels of trypsin and aminopeptidase N (APN) in the intestinal mucosa showed their highest levels (*P* < 0.05) in the NRC100 and AC treatments. The mRNA levels of trypsinogen and APN were significantly up-regulated (*P* < 0.05) in the AC, CN, and NRC100 treatments compared with the control. The mRNA levels for the Zn transporter genes SLC30A1 (ZnT1) and SLC30A2 (ZnT2) were significantly up-regulated (*P* < 0.05) in the AC treatment, and the mRNA levels for SLC39A4 (ZIP4) and metallothionein 1 (MT) in the AC, CN, and NRC100 treatments were significantly up-regulated (*P* < 0.05) compared with the control. Meat quality were not affected (*P* > 0.05) by the different recommended levels of Cu and Zn. These results indicated that the supplemental Cu and Zn levels routinely used in AC diets in Chinese commercial feed enterprises should be reduced.

## Introduction

Copper (Cu) and zinc (Zn) are essential mineral elements for all animals, including pigs and poultry, and often need to be supplemented in commercial feeds to prevent post-weaning diarrhea and as an alternative to in-feed antibiotics to stimulate growth and immune response (1–4). Those minerals are also essential components of several metalloenzyme systems, and Cu is surpassed only by Zn in the numbers of enzymes that it can activate for various biochemical programs, including normal growth and development of organs and the immune system (1, 4–6). The previous (1998) and current (2012) National Research Council (NRC) publications state the amounts of Cu and Zn required by pigs as 3.5 and 50 mg/kg dry diet, respectively, and further describe essential micromineral requirements on a total basis, stating that requirements include the contributions of all dietary ingredients (9–13), however, the current NRC publication (2012) also indicated that micromineral levels and bioavailability are variable and largely unknown (10, 13). Pig diets commonly contain these mineral elements in amounts that exceed the 2012 NRC-recommended levels (12), and any amounts that pigs consume in excess of their dietary requirements are excreted through feces into the environment, resulting in environmental issues (9, 14). In China, the Zn and Cu levels in pig diets are 14.48 and 1.67 times higher, respectively, than the levels recommended by the Ministry of Agriculture of the People's Republic of China (MA-PRC) (7), and the First National Census on Pollution also indicated that 2.4 × 10^3^ tons of Cu and ×10^3^ tons of Zn are discharged as major water pollutants from the livestock industry (7). The commercial pig industry in China must confront its potential impact on environmental pollution, as 3.5 mg/kg of Cu and 150 mg/kg of Zn are often added in practice to commercial pig diets by the Chinese feed industry (Table 1) (7, 9), but the current data on fecal Cu and fecal Zn excreted from pigs that receive those diets are estimated and remain unclear for the most part (7, 9). An effective strategy for reducing Cu and Zn excreted into the soil and water is for pig diets to include organic Cu and Zn mineral sources that may exhibit greater bioavailability than commonly used inorganic sources of the same minerals, and numerous studies have indicated that chelated sources of Cu and Zn are more bioavailable than inorganic sources of those minerals (1, 4, 9, 11, 14, 15). Limited data are available on the use of zinc-methionine chelate (Zn-Met) and cupreous N-carbamylglutamate chelate (NCG-Cu) as the only sources of Zn and Cu at NRC-recommended, MA-PRC-recommended, and actual Chinese feed industry levels in diets for finishing pigs.

**Table 1:** The recommended levels of Cu and Zn in pig feeds in different countries and the actual amounts used in the Chinese feed industry.

**Concentrations**		**Ranges of body weight (kg)**			
		**2–12**	**12–30**	**30–70**	**70–110**
Chinese recommendations (mg/kg)^a^	Cu	3–6	3–6	3–6	3–6
	Zn	43–120	43–120	43–120	43–120
Amounts actually used in the Chinese feed industry (mg/kg)^b^	Cu	250	200	150	35
	Zn	3,000	150	150	150
		**8–28**	**28–60**	**60–110**	
NRC (1998) (mg/kg)^c^	Cu	6.0–5.0	4.0	3.5	
	Zn	100–80	60	50	
INRA (1989) (mg/kg)^d^	Cu	10	10	10	
	Zn	100	100	100	
ARC (1981) (mg/kg)^e^	Cu	3.6	3.6	3.6	
	Zn	45	45	45	
GfE (1987) (mg/kg)^f^	Cu	5.4	4.5	4.5	
	Zn	90–72	54	45	

a *Levels recommended in Bulletin No. 1224 of the Ministry of Agriculture of the People's Republic of China.*

b *Actuallevels used in Chinesecommercial feed enterprises; data adapted from Tan et al. (7).*

c *NRC (8).*

d *INRA, 1989; data adapted from Jondreville et al. (9).*

e *ARC, 1981, data adapted from Jondreville et al. (9).*

f *GfE, 1987; data adapted from Jondreville et al. (9)*.

Previous studies have shown that Cu and Zn can influence digestive enzyme activity and metal transporter gene expression in weanling pigs and fish (6, 16–19), but pigs at different life stages have shown adaptations in enzyme secretion expression levels in response to dietary changes (6, 18, 20–23). Additionally, a high Zn supply (3,000 mg of Zn/kg of diet) leads to down-regulation of ZIP4 and ZnT1 in the jejunum of growing rats (24), and high Cu (225 mg of Cu/kg of tribasic Cu chloride (TBCC) or CuSO_4_ diet) also modulates the expression of Atox1in the liver Atp7b in the duodenum of weanling pigs (25). However, very few data are yet available in humans or their omnivorous animal model, the pig (19, 26). In general, finishing pigs produce and excrete more feces than weanling pigs do (7, 10, 27), and the digestive, absorptive, and transportational abilities of finishing pigs can also affect the amounts of Cu and Zn excreted into the soil and water when Cu and Zn are added to diets at high concentrations, as is often the case (2, 7, 9, 10, 22, 27).

This study investigated the effect of dietary supplementation with Zn-Met and NCG-Cu at the NRC (2012), MA-PRC, and actual Chinese feed industry levels on antioxidant capacity, tissue mineral status, mineral excretion, meat quality, digestive enzyme activity, and metal transporters in finishing pigs.

## Materials and Methods

### Animals

A total of 120 finishing pigs (Yorkshire × Landrace) with an average initial body weight (BW) of 70.0 ± 2.1 kg were allotted by sex, weight, and litter to different dietary treatments and fed to a final body weight (BW) of ~110 kg. The pigs were the progeny of Yorkshire × Landrace sows (Hunan Baodong Farming Development Co., Ltd., Shaoyang, China).

### Treatments, Diet Composition, and Animal Housing

The four treatments consisted of (1) basal diet without added Cu and Zn (control), (2) basal + 35 mg of supplemental Cu/kg from NCG-Cu + 150 mg of supplemental Zn/kg from Zn-Met (actual levels used in China commercial feed enterprises) (AC), (3) basal + 3.0 mg of supplemental Cu/kg from NCG-Cu + 43 mg of supplemental Zn/kg from Zn-Met (minimum recommended levels in Bulletin No. 1224 of the Ministry of Agriculture of the People's Republic of China) (CN), and (4) basal + 3.5 mg of supplemental Cu/kg from NCG-Cu + 50 mg of supplemental Zn/kg from Zn-Met (100% of NRC requirements for Cu and Zn) (NRC100) (Table 2). The amounts of Cu and Zn added to the basal diet, and the analytically determined intrinsic mineral content of the basal diets are shown in Table 2. NCG-Cu and Zn-Met were purchased from Tanke (Tanke Bio-Technology Co., Ltd., Guangzhou, China) and XJ (XJ Bio-Technology Co., Ltd., Changsha, China), respectively. Pigs had *ad libitum* access to feed and water. All diets met the nutritional requirements for pigs according to the National Research Council (Table 3) (12). Growth performance was measured in terms of average daily gain (ADG), average daily feed intake (ADFI), and feed-to-gain ratio (F/G) (1, 28, 29).

**Table 2:** Amounts of Cu and Zn added to the basal diet; analytically determined intrinsic mineral content in the basal diet.

	**Dietary supplementation**			
		**Actual levels used by Chinese feed enterprises**	**Chinese reference levels**	**100%NRC (1998) levels**
**Items**	**Control^a^**	**AC^b^**	**CN^c^**	**NRC100^d^**
**Added to the basal diet (mg/kg)**				
NCG–Cu	0	35	3.0	3.5
Zn-Met	0	150	43	50
**Analytically determined intrinsic minerals (mg/kg of DM)**				
	**Cu**	**Zn**	**Fe**	**Mn**
Basal diet^e^	7.84 ± 1.23	45.18 ± 11.34	248.49 ± 9.34	25.83 ± 2.23

*NCG-Cu, cupreous N-carbamylglutamate chelate; Zn-Met, zinc-methionine chelate; DM, dry matter.*

a *Control = basal diet (basal diet without added Cu or Zn).*

b *AC = basal diet + 35 mg of supplemental Cu/kg from NCG-Cu + 150 mg of supplemental Zn/kg from Zn-Met (actual levels used by Chinese commercial feed enterprises).*

c *CN = basal diet + 3.0 mg of supplemental Cu/kg from NCG-Cu + 43 mg of supplemental Zn/kg from Zn-Met (minimum recommended levels in Bulletin No. 1224 of the Ministry of Agriculture of the People's Republic of China).*

d *NRC100 = basal diet + 3.5 mg of supplemental Cu/kg from NCG-Cu + 50 mg of supplemental Zn/kg from Zn-Met (100% of NRC levels of Cu and Zn requirements).*

e *Mean values ± SD (n = 8)*.

**Table 3:** Compositions and nutrient levels in basal diets (as-fed basis).

**Ingredients**	**Contents (%)**	**Calculated and analytically determined nutrient composition^b^**	**Contents**
Corn (43%CP)	67.00	DE (MJ/kg)^c^	14.20
Soybean meal	23.76	CP	16.30
Wheat bran	6.00	Total Ca	0.52
Soybean oil	0.88	Total P	0.45
Lysine hydrochloride	0.01	Starch	43.71
Hydroxy methionine	0.00	NDF	11.33
L-threonine	0.00	ADF	4.34
L-tryptophan	0.00	Lys	0.72
CaHPO_3_	0.50	Met + Cys	0.50
Rock powder	0.55	Thr	0.56
Salt	0.30	Trp	0.17
1% premix^a^	1.00	Arg	0.94
Total	100.00	His	0.39
EAA	6.29	Ile	0.60
NEAA	8.76	Leu	1.32
EAA/NEAA	0.70	Phe	0.71
		Val	0.61

a *The premix provided the following amounts of vitamins and minerals per kilogram on an as-fed basis: vitamin A (acetate), 1,500 IU; vitamin D_3_, 300 IU; vitamin E, 15 IU; vitamin K (menadione), 0.5 mg; riboflavin, 10 mg; D-pantothenic acid, 13 mg; niacin, 10 mg; folacin, 0.4 mg; D-biotin, 1 mg; vitamin B_12_, 15 μg; D-calcium pantothenate, 25 mg; Fe, 50 mg as ferrous sulfate; Mn, 2 mg as manganese oxide; I, 0.5 mg as potassium iodide; and Se, 0.3 mg as sodium selenite. The values are expressed as percentages (%) except for digestible energy (DE; MJ/kg), and essential amino acids (EAA)/non-essential amino acids (NEAA); CP, crude protein; NDF, neutral detergent fiber; ADF, acid detergent fiber.*

b *All other values represent analytically determinedvalues.*

c *The DE was calculated according to the NRC (2012)*.

### Sample Collection

At the end of the experiment (day 52), 8 pigs/treatment (4 barrows and 4 gilts) were randomly selected and fasted overnight (for ~12 h), then sacrificed by electrical stunning (250 V, 0.5A, for 5~6s) (1, 4, 28, 29). The liver, spleen, kidney, heart, and longissimus dorsi (LD) were removed, collected, and weighed as previously described (1, 28, 29). Fecal grab samples were obtained by rectal palpation from 8 pigs per treatment for fecal mineral analysis, and the blood and tissue samples for each treatment were separated and stored as previously described (1, 28, 29). The midpoint of the duodenum mucosa was harvested and collected in sterilized plastic bottles in an ice box for subsequent analysis of the protein levels of digestive enzymes (28). Additionally, segments (~1–2 cm) surrounding the midpoint of each duodenum were immediately flushed 4 times with ice-cold phosphate-buffered saline (PBS), rapidly frozen in liquid nitrogen, and stored at −80°C for subsequent analysis of gene expression (22, 30).

### Measurement of Serum Antioxidant Capacity

Serum superoxide dismutase (SOD), glutathione peroxidase (GSH-P*x*), malondialdehyde (MDA), glutathione (GSH), total antioxidant capacity (T-AOC), and Cu/Zn superoxide dismutase (Cu/Zn SOD) were measured with a commercial kit (Nanjing Jiancheng Bioengineering Institute, Nanjing, China) and a Beckman CX4 Chemistry Analyzer (Beckman Coulter, Brea, CA) (28, 29, 31).

### Analysis of Organ Mineral Status

The mineral levels in the feed, feces, liver, spleen, and kidney were measured by inductively coupled plasma optical emission spectrometry (ICP-OES) (Agilent 7700, Agilent, Santa Clara, CA, USA) as described previously (1, 4).

### Measurement of Digestive Enzyme Activity

Samples of the duodenum mucosa were homogenized in ice-cold PBS and then centrifuged at 10,000 × g at 4°C for 10 min. The supernatant was collected, and the lactase, maltase, sucrase, trypsin, lipase, and aminopeptidase N (APN) levels were measured using ELISA kits (Nanjing Jiancheng Bioengineering Institute, Nanjing, China) and Beckman CX4 Chemistry Analyzer (Beckman Coulter, Brea, CA)as described previously (6, 22).

### RNA Isolation and Quantitative Real-Time PCR

Total RNA isolation and quantitative real-time PCR were performed in duodenum intestinal mucosa using a previously described method (28, 29, 31). Real-time PCR primers were designed and selected on the basis of published sequences (Table 4) (18, 19, 22, 23, 32). Genes targeted in the intestinal mucosal sections included digestive enzyme genes (pepsinogen A, trypsinogen, chymotrypsin C, lactase, aminopeptidase N (APN), lipase, sucrase, maltase, and amylase), Zn transporter genes [zinc transporter SLC30A1 (ZnT1), zinc transporter SLC30A2 (ZnT2), zinc transporter SLC30A5 (ZnT5), zinc transporter SLC39A4 (ZIP4), divalent metal transporter 1 (DMT1), and metallothionein 1 (MT)], and Cu transporter genes [Cu transporter 1 (Ctr1), antioxidant 1 (Atox1), Cu transporting α-polypeptide ATPase (Atp7a), Cu transporting β-polypeptide ATPase (Atp7b), and cytochrome c oxidase assembly protein 17 (Cox17)]. Quantitative real-time PCR reactions were run on an ABI 7900 PCR system (ABI Biotechnology, Eldersburg, MD, USA), and each reaction had a total volume of 25 μL, containing 12.5 μL of SYBR Green mix and 1 μmol/L each of forward and reverse primers. The thermal cycling program was run as previously reported (28, 29, 31). Previously reported data were used to assess numerous housekeeping genes for their stability in intestinal tissue (32). Of those assessed, TATA box binding protein (TBP1), ribosomal protein L4 (RPL4), hypoxanthine phosphoribosyl transferase 1 (Hprt1), and beta-actin (β-actin) were found to be the most stable. The relative expression of target genes was determined using 2^−(Δ*ΔCt*)^as previously reported (28, 29, 31).

**Table 4:** Primers used for relative quantitative PCR analysis.

**Target gene**	**Primer sequence**	**GenBank accession number**	**Size (bp)**	***T***_***M***_ **(°C)**	**References**
**Digestive enzyme genes**				
Pepsinogen A	Sense 5′-TGTTTCCGTCGAGGGTTACTG-3′	NM_213873.2	159	60.0	(23)
	Antisense 5′- CCGATGTCGCTCTGGATGTT-3**'**				
Trypsinogen	Sense 5′ -AGCAATTCATCAATGCCGCC-3′	NM_001162891.1	253	59.0	(22)
	Antisense 5′- CAGGAGCGAAGGGTAGCTG-3′				
Chymotrypsin C	Sense 5′-GCGGCACCTTAATCACCTCT-3′	NM_001244379.2	101	60.0	(22)
	Antisense 5′-GGCAGGCATAACACCTGGAT-3′				
Lactase	Sense 5′-GCTACCACCTAATACAGTAAACCTCCC-3	XM_003359430	235	55.0	(23)
	Antisense 5′-CCCACAGAAAGTCATCCCGAAA-3′				
Aminopeptidase N	Sense 5′-TCATCAATCGGGCTCAGGTC-3′	HQ824547.1	101	55.0	(23)
	Antisense 5′-TCCGTTCAGGAAGAGGGTGTT-3′				
Lipase	Sense 5′-AAGGTGGAGAGCGTGAACTG-3′	NM_001177912.2	205	55.0	(22)
	Antisense 5′-TCCAGCCCTGTGATTCGTTC-3′				
Sucrase	Sense 5′-TGGTGGCACTGTTATCCGAC-3′	XM_005657098.1	166	55.0	(22)
	Antisense 5′-GAGCAGGCTCTTGACATGGT-3′				
Maltase	Sense 5′-GCACAGATCAGCCGATGAGA-3′	XM_005657730.1	154	62.0	(22)
	Antisense 5′-CAAATGACCGTCCAGCTCCT-3′				
Amylase	Sense 5′-GGGCAGCGTTTATTCTCACTCA-3′	NM_214195	252	65.0	(23)
	Antisense 5′-TCTCTTGCTTCTTCCCTGTTCC-3'				
**Zinc transporter genes**
ZnT1	Sense 5′-CCAGGGGAGCAGGGAACCGA-3′	NM_001139470.1	73	60.0	(18)
	Antisense 5′-TCAGCCCGTTGGAGTTGCTGC-3′				
ZnT2	Sense 5′-GACAGCGCCAGCCAGCATCA-3′	NM_001139475.1	99	60.0	(18)
	Antisense 5′-GGCAGCCACCAAAACGCCCA-3′				
ZnT5	Sense 5′-ACCAGTCTCAGTTGGAGGGCTGA-3′	NM_001137624.1	79	60	(18)
	Antisense 5′-TCCATGGGTATGGGTGTGGGCA-3′				
ZIP4	Sense 5′- TGCTGAACTTGGCATCTGGG−3′	XM_021090449.1	125	60	(18)
	Antisense 5′- CGCCACGTAGAGAAAGAGGC-3′				
DMT 1	Sense 5′-CGCGCTTCGCCCGAGTGAT−3′	XM_021081710.1	70	60	(18)
	Antisense 5′-TGGAAGACGGCCACCAGCAGA-3′				
MT	Sense 5′-GTGAATCCGCGTTGCTCTCTGCT-3′	XM_021093891.1	72	60	(18)
	Antisense 5′-CTGTGGGGCAGGAGCAGTTGG−3′				
**Copper transporter genes**
Ctr1	Sense 5′-ATGATGATGATGCCTATGACC-3′	NM214100.2	150	60	(17)
	Antisense 5′-GATGCTGACTTGGGACTTG-3′				
Atox1	Sense 5′-CCGAAGCACGAGTTCTCC-3′	NM001167641.1	109	58.0	(17)
	Antisense 5′-TGTTGGGCAGGTCAATGTC-3′				
Atp7a	Sense 5′-AAGGAGGAGACAAAGACTTCATC-3′	AB271958.1	200	57.5	(17)
	Antisense 5′-CGGATTAACTCTGCTATCATCAAG-3′				
Atp7b	Sense 5′-TCACTAAGAAGCCTGGAAG-3′	XM001925351.1	148	55.0	(17)
	Antisense 5′-ATGGGTGCCTTTGACATC-3′				
Cox17	Sense 5′-GAGCACTGTGGACACCTAATTGAG-3′	NM001190922.1	86	61.0	(17)
	Antisense 5′-TCACAACGCAGACCACCATTTC-3′				
**Housekeeping genes**
β-actin	Sense 5′-CACGCCATCCTGCGTCTGGA-3′	DQ845171	100	63.0	(32)
	Antisense 5′-AGCACCGTGTTGGCGTAGAG-3′				
Rpl4	Sense 5′-CAAGAGTAACTACAACCTTC-3′	DQ845176	122	60.0	(32)
	Antisense 5′-GAACTCTACGATGAATCTTC-3′				
TBP1	Sense 5′-AACAGTTCAGTAGTTATGAGCCAGA-3′	DQ845178	153	60.0	(32)
	Antisense 5′-AGATGTTCTCAAACGCTTCG-3′				
HMBS2	Sense 5′-AGGATGGGCAACTCTACCTG-3′	DQ845175	83	58.0	(32)
	Antisense 5′-GATGGTGGCCTGCATAGTCT-3′				

*T_M_, melting temperature; ZnT1, zinc transporter SLC30A1; ZnT2, zinc transporter SLC30A2; ZnT5, zinc transporter SLC30A5; ZIP4, zinc transporter SLC39A4; DMT 1, divalent metal transporter 1; MT, metallothionein 1; Ctr1, Cu transporter 1; Atox1, antioxidant 1; Atp7a, Cu transporting α-polypeptide ATPase; Atp7b, Cu transporting β-polypeptide ATPase; Cox17, cytochrome c oxidase assembly protein 17; Rpl4, ribosomal protein L4; TBP1, TATA box binding protein 1; HMBS2, hydroxymethylbilane synthase 2*.

### Measurement of Meat Quality

Pre-slaughter BW, carcass weight, carcass length, backfat thickness, and LD muscle area were immediately measured according to previously reported methods (33). Samples of the LD muscle were taken from the area of the last thoracic vertebra to analyze the quality of the carcasses. Meat acidity was measured with a pH meter (Matthaus pH Star, Germany) 45 min and 24 h after slaughter (34). Meat color traits including lightness (L^*^), redness (a^*^), and yellowness (b^*^) were measured at 24 h postmortem in LD samples at the last lumbar vertebra using a hand-held colorimeter (CR-410, Konica Minolta Sensing Inc., Osaka, Japan); the measurements were standardized against a white calibration plate (34). Marbling scores (ranging from 0 to 3, with 0 = absent and 3 = overly abundant) were subjectively evaluated according to previously reported methods (34). The shear force was determined using a Warner-Bratzler shear force device (TA.XT. Plus, Stable Micro Systems, Godalming, UK) in samples cooled at 4°C for 24 h after heat treatment, according to previously reported methods (34).

### Statistical Analysis

Data analysis was performed by analysis of variance (ANOVA) using the MIXED procedure in SAS (Version 8.2; SAS Inst. Inc., Cary, NC) with pen (*n* = 8) as the experimental unit (1, 2, 28, 29). The model for all data (including growth performance, serum antioxidant activity, digestive enzyme protein activity, organ mineral status, and meat quality parameters) included a fixed effect of treatment and random effects. Data were subjected to the HOVTEST option in SAS to account for homogeneity of variance and normality. In addition, data obtained from RT-PCR measurements were analyzed by multifactor ANOVA using the GLM procedure to assess the effects of different treatments. The gene expression results of the statistical analyses were visualized with GraphPad Prism 6.0 software (GraphPad Software, Inc., San Diego, CA). Data are expressed as the mean ± SEM. Values in the same row with different superscripts are significant (*P* < 0.05).

## Results

### Growth Performance

Growth performance is shown in Table 5. For the overall experimental period, there were no significant effects (*P* > 0.05) of different recommended levels of Cu and Zn in combination on ADG, ADFI, or G/F.

**Table 5:** The effect of dietary supplementation with different levels of Cu and Zn on the growth performance of finishing pigs.

	**Dietary supplementation**					
**Items**	**Control^**1**^**	**AC^**2**^**	**CN^**3**^**	**NRC100^**4**^**	**SEM ±**	***P* value**
Initial BW, kg	71.12	72.23	71.32	72.37	0.735	0.627
Final BW, kg	112.12	114.22	112.31	113.54	1.546	0.794
ADG, kg/d	0.804	0.822	0.811	0.815	0.044	0.723
ADFI, kg/d	2.60	2.65	2.64	2.66	0.053	0.697
F/G	3.24	3.23	3.25	3.27	0.074	0.667

*^a, b, c^Means within a row that do not have a common superscript letter are significantly different (P <0.05). NCG-Cu, cupreous N- carbamylglutamate chelate; Zn-Met, zinc-methionine chelate. ^1^Control = basal diet (without added Cu or Zn). ^2^AC = basal diet + 35 mg of supplemental Cu/kg from NCG-Cu + 150 mg of supplemental Zn/kg from Zn-Met (actual levels used by Chinese commercial feed enterprises). ^3^CN = basal diet + 3.0 mg of supplemental Cu/kg from NCG-Cu + 43 mg of supplemental Zn/kg from Zn-Met (minimum recommended levels in Bulletin No. 1224 of the Ministry of Agriculture of the People's Republic of China). ^4^NRC100 = basal diet + 3.5 mg of supplemental Cu/kg from NCG-Cu + 50 mg of supplemental Zn/kg from Zn-Met (100% of NRC requirements for Cu and Zn); ADG, average daily gain; ADFI, average daily feed intake; F/G, feed-to-gain ratio*.

### Serum Antioxidant Capacity

Serum antioxidant capacity is shown in Table 6. Among the four treatments, AC had the highest level (*P* < 0.05) of GSH-P*x*, and there were no significant differences between the AC and CN treatments, but there was significant difference between the AC and NRC100 treatments.Pigs fed the NRC100 diet (3.5 mg/kg NCG-Cu+ 50 mg/kg Zn-Met) tended to have higher levels (*P* = 0.063) of T-AOC than those receiving the other treatments, but the difference was not significant.Pigs fed the AC diet (35 mg/kg NCG-Cu + 150 mg/kg Zn-Met) tended to have higher levels (*P* = 0.074) of Cu/Zn SOD than those receiving the other treatments, but the difference was not significant. No effects (*P* > 0.05) of treatment were detected on the levels of SOD, MDA, GSH, or T-AOC.

**Table 6:** The effects of dietary supplementation with different levels of Cu and Zn on serum antioxidant activity in finishing pigs (*n* = 8).

	**Dietary supplementation**					
**Items**	**Control^**1**^**	**AC^**2**^**	**CN^**3**^**	**NRC100^**4**^**	**SEM ±**	***P-*value**
**Antioxidant activity**						
SOD (U/ml)	107.27	120.79	118.64	109.55	5.483	0.326
GSH-P*x* (U/ml)	387.46^c^	552.69^b^	525.73^b^	452.08^a^	47.597	0.043
MDA (nmol/ml)	7.48	7.91	7.63	7.86	0.213	0.134
GSH (μmol/L)	231.24	252.97	243.45	255.74	51.272	0.623
T-AOC (U/ml)	1.623	1.798	0.674	1.834	0.376	0.063
Cu/Zn SOD (U/ml)	221.14	298.43	235.62	294.67	48.762	0.074

*^a, b, c^Means within a row that do not have the same superscript letter are significantly different (P <0.05). NCG-Cu, cupreous N- carbamylglutamate chelate; Zn-Met, zinc-methionine chelate. ^1^Control = basal diet (without added Cu or Zn). ^2^AC = basal diet + 35 mg of supplemental Cu/kg from NCG-Cu + 150 mg of supplemental Zn/kg from Zn-Met (actual levels used by Chinese commercial feed enterprises). ^3^CN = basal diet + 3.0 mg of supplemental Cu/kg from NCG-Cu + 43 mg of supplemental Zn/kg from Zn-Met (minimum recommended levels in Bulletin No. 1224 of the Ministry of Agriculture of the People's Republic of China). ^4^NRC100 = basal diet + 3.5 mg of supplemental Cu/kgfrom NCG-Cu + 50 mg of supplemental Zn/kg from Zn-Met (100% of NRC requirements for Cu and Zn). SOD, superoxide dismutase; GSH-Px, glutathione peroxidase; MDA, malondialdehyde; GSH, glutathione; T-AOC, total antioxidant capacity; Cu/Zn SOD, Cu/Zn superoxide dismutase*.

### Organ Mineral Status and Fecal Minerals Excretion

The mineral levels in the organs (liver, kidney, and heart) and feces are listed in Table 7. Pigs fed the AC diet had the highest (*P* < 0.05) liver Zn among the four treatments, but the levels did not differ between those fed the AC and CN diets. The Cu, Zn, Fe, and Mn levels in the kidney and heart did not differ (*P* > 0.05) among the four treatments.

**Table 7:** The effects of dietary supplementation with different levels of Cu and Zn on mineral levels in organs and feces (mg/kg of DM) (*n* = 8).

	**Dietary supplementation**					
**Items**	**Control^**1**^**	**AC^**2**^**	**CN^**3**^**	**NRC100^**4**^**	**SEM ±**	***P-*value**
**Liver mineral levels**						
Cu	5.78	6.11	5.90	5.82	0.417	0.622
Zn	74.13^a^	89.37^b^	81.07^b^	78.26^a^	5.836	0.041
Fe	265.13	270.33	272.56	274.59	198.66	0.878
Mn	4.18	4.23	4.15	4.18	0.273	1.242
**Kidney mineral levels**						
Cu	6.11	5.72	5.94	5.69	0.457	0.974
Zn	30.15	33.79	31.68	34.69	3.273	1.227
Fe	41.22	45.29	43.96	47.37	4.286	0.683
Mn	1.32	1.57	1.39	1.58	0.271	1.544
**Heart mineral levels**						
Cu	3.24	3.98	3.73	4.03	0.156	2.574
Zn	18.04	20.38	18.36	20.17	1.615	2.331
Fe	42.13	46.93	45.78	46.01	1.873	0.976
Mn	1.47	1.41	1.57	1.40	0.212	1.281
**Fecal mineral levels**						
Cu	110.13^a^	158.49^b^	120.44^c^	125.27^a^	42.374	0.042
Zn	461.37^a^	522.44^b^	481.69^c^	486.73^c^	38.177	0.038
Fe	1213.27	1437.18	1374.36	1329.61	236.552	0.0673
Mn	411.37	435.27	424.31	429.69	43.173	0.137

*^a, b, c^Means within a row without a common superscripted letter are significantly different (P <0.05). DM, dry matter; NCG-Cu, cupreous N- carbamylglutamate chelate; Zn-Met, zinc-methionine chelate.^1^Control = basal diet (without added Cu or Zn). ^2^AC = basal diet + 35 mg of supplemental Cu/kg from NCG-Cu + 150 mg of supplemental Zn/kg from Zn-Met (actual levels used by Chinese commercial feed enterprises). ^3^CN =basal diet + 3.0 mg of supplemental Cu/kg from NCG-Cu + 43 mg of supplemental Zn/kg from Zn-Met (minimum recommended levels in Bulletin No. 1224 of the Ministry of Agriculture of the People's Republic of China). ^4^NRC100 = basal diet + 3.5 mg of supplemental Cu/kg from NCG-Cu + 50 mg of supplemental Zn/kg from Zn-Met (100% of NRC requirements for Cu and Zn)*.

Fecal Cu levels increased (*P* < 0.05) as the levels of Cu and Zn in the feed increased. The levels of fecal Cu and Zn were highest (*P* < 0.05) when 35 mg/kg NCG-Cu and 150 mg/kg Zn-Met (AC treatment) were fed, but there was no difference (*P* > 0.05) between the NRC100 and control treatments except in terms of fecal Zn levels.

### Digestive Enzyme Protein Activity and mRNA Expression Levels

**Figure 1:**
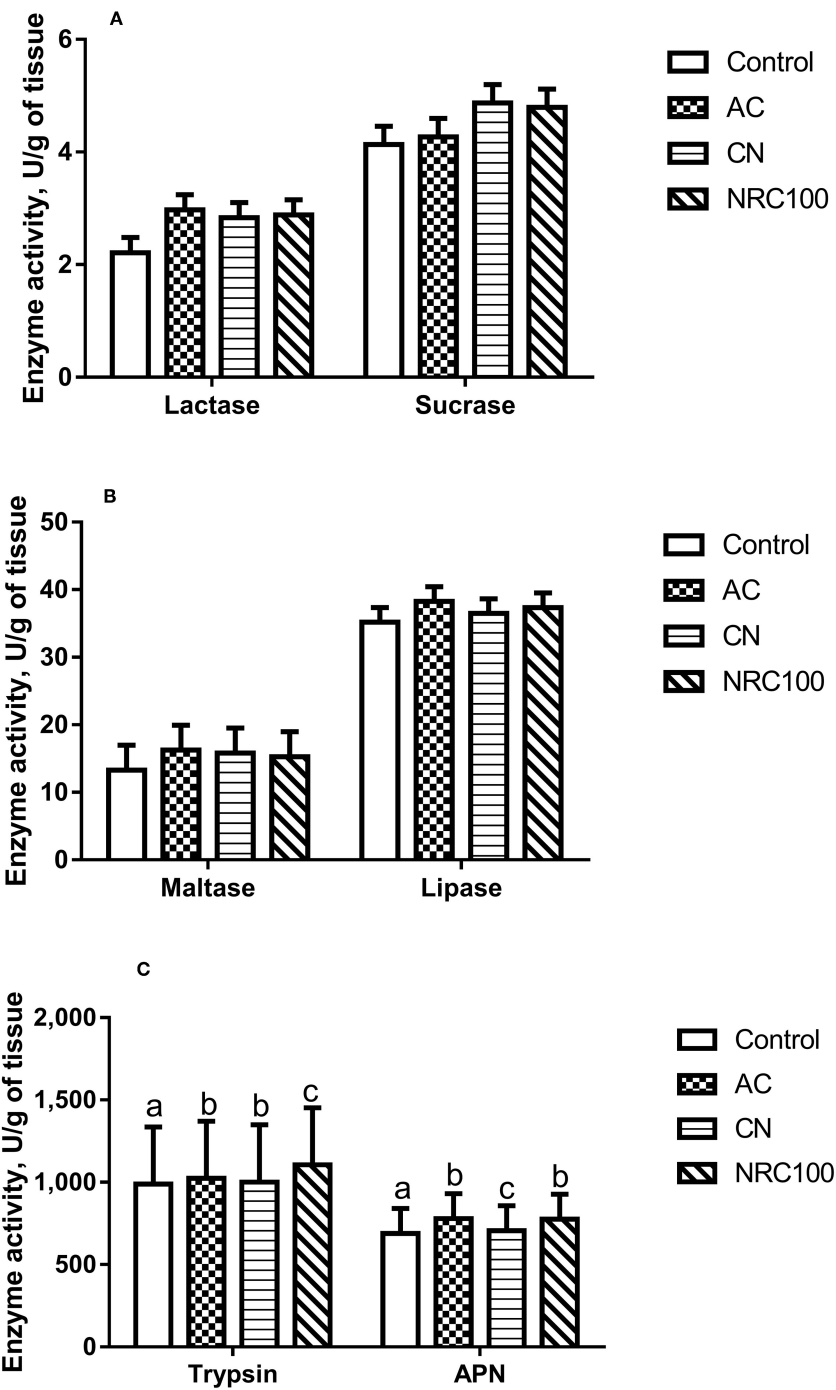
Effects of different levels of Cu and Zn on digestive enzyme protein activity in the small intestine mucosa (U/g of tissue) of finishing pigs (*n* = 8). NCG- Cu, cupreous N-carbamylglutamate chelate diet; Zn-Met, zinc-methionine chelate; Control, basal diet (basal diet without added Cu or Zn); AC, basal diet + 35 mg of supplemental Cu/kg from NCG-Cu + 150 mg of supplemental Zn/kg from Zn-Met (actual levels used in the Chinese commercial feed industry); CN, basal diet + 3.0 mg of supplemental Cu/kg from NCG-Cu + 43 mg of supplemental Zn/kg from Zn-Met (minimum recommended levels in Bulletin No. 1224 of the Ministry of Agriculture of the People's Republic of China); NRC100, basal diet + 3.5 mg of supplemental Cu/kg from NCG-Cu + 50 mg of supplemental Zn/kg from Zn-Met (100% of NRC requirements for Cu and Zn). **(A)** Lactase and sucrose protein activity; **(B)** maltase and lipase protein activity; **(C)** trypsin and amino peptidase N (APN) protein activity. ^a, b, c^ Means within a row without a common superscripted letter are significantly different (*P* < 0.05).

The protein levels of digestive enzymes in duodenum intestinal mucosa are shown in Figure 1. The protein level of trypsin was higher in the NRC100 group (*P* < 0.05) than in any of the other treatment groups, and there were no significant differences between the AC and CN treatments. The protein level of APN was higher in the AC group (*P* < 0.05) than in the other groups, and there were no significant differences between the AC and NRC100 treatments. The protein levels of lactase, sucrase, maltase, and lipase did not differ (*P* > 0.05) among the four diet treatments.

**Figure 2:**
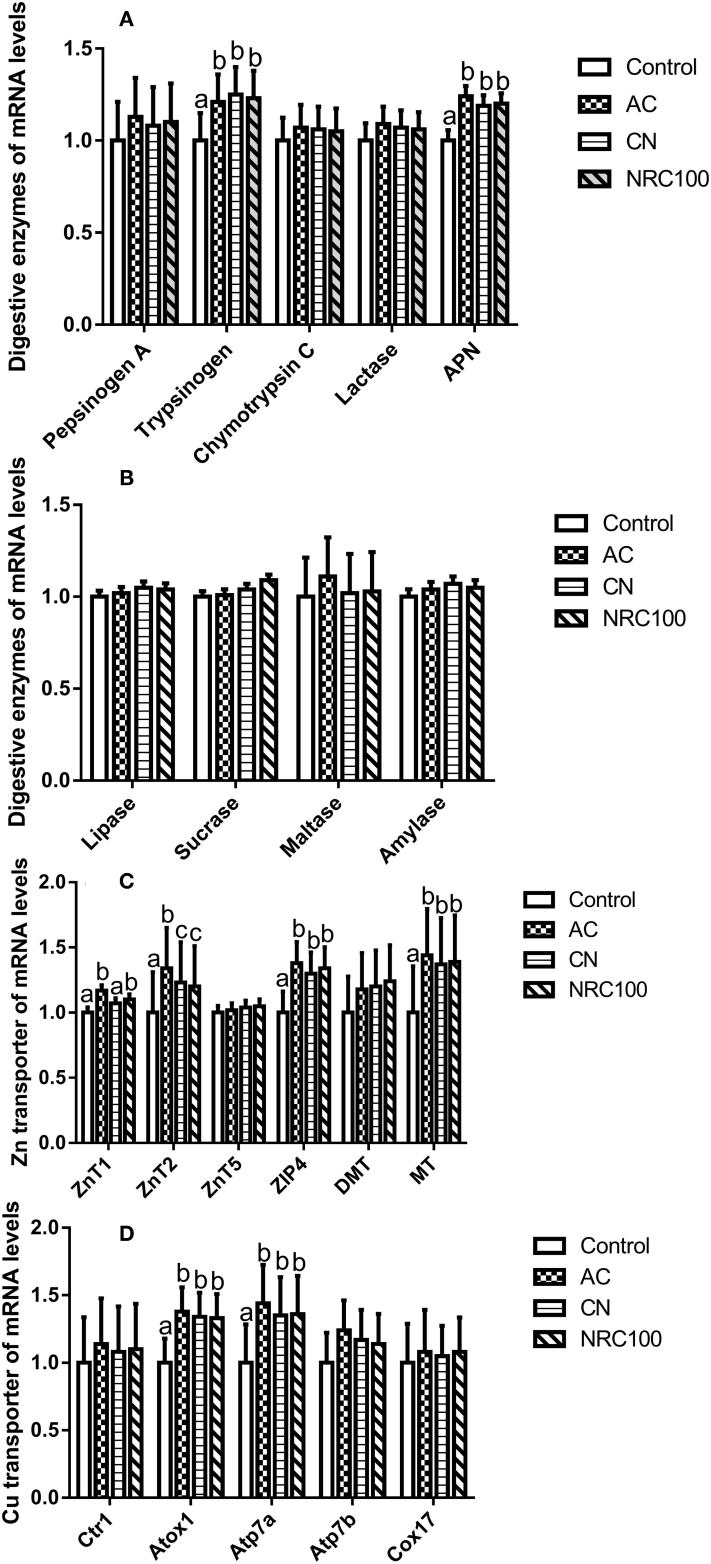
Relative mRNA levels of Cu and Zn transporter genes and digestive enzyme genes in the small intestine mucosa (U/g of tissue) of finishing pigs (*n* = 8). NCG-Cu, cupreous N-carbamylglutamate chelate diet; Zn-Met, zinc-methionine chelate; Control, basal diet (basal diet without added Cu or Zn); AC, basal diet + 35 mg of supplemental Cu/kg from NCG-Cu + 150 mg of supplemental Zn/kg from Zn-Met (actual levels used in the Chinese commercial feed industry); CN, basal diet + 3.0 mg of supplemental Cu/kg from NCG-Cu + 43 mg of supplemental Zn/kgfrom Zn-Met (minimum recommended levels in Bulletin No. 1224 of the Ministry of Agriculture of the People's Republic of China); NRC100, basal diet + 3.5 mg of supplemental Cu/kg from NCG-Cu + 50 mg of supplemental Zn/kg from Zn-Met (100% of NRC requirements for Cu and Zn). **(A)** mRNA expression levels of the genes encoding the digestive enzymes pepsinogen A, trypsinogen, chymotrypsin C, lactase, and amino peptidase N (APN); **(B)** mRNA expression levels of the genes encoding the digestive enzymes lipase, sucrose, maltase, and amylase; **(C)** mRNA expression levels of the genes encoding the zinc transportersSLC30A1 (ZnT1), SLC30A2 (ZnT2), SLC30A5 (ZnT5),SLC39A4 (ZIP4), divalent metal transporter 1 (DMT1), and metallothionein 1 (MT); and **(D)** mRNA expression levels of the genes encoding the copper transporter proteins Cu transporter 1 (Ctr1), antioxidant 1 (Atox1), Cu^2+^-transporting α-polypeptide ATPase (Atp7a), Cu^2+^-transporting β-polypeptide ATPase (Atp7b), and cytochrome c oxidase assembly protein 17 (Cox17). ^a, b, c^ Means within a row without a common superscripted letter are significantly different (*P* < 0.05).

The mRNA levels for trypsinogen and APN were significantly up-regulated (*P* < 0.05) in the AC, CN, and NRC100 diet treatments compared with the control diet, but there was no difference among the AC, CN, and NRC100 treatments (Figure 2A).Apart from those mentioned above, the mRNA levels for digestive enzymes remained unaffected by the four diet treatments (*P* > 0.05) (Figures 2A,B).

### mRNA Expression Levels of Cu and Zn Transporter Genes

The mRNA expression levels of Zn transporter genes (ZnT1, ZnT2, ZIP4, and MT) were affected by the dietary treatments (Figure 2C). The mRNA level for ZnT1 was significantly up-regulated by the AC treatment (*P* < 0.05) compared with the control and CN treatments, but there was no difference (*P* > 0.05) between the AC with NRC100 treatments. The mRNA level for ZnT2 was significantly up-regulated by the AC treatment (*P* < 0.05) compared with the other treatments, but there was no difference (*P* > 0.05) between the CN and NRC100 treatments. The mRNA levels for ZIP4 and MT were significantly up-regulated by the AC, CN, and NRC100 treatments (*P* < 0.05) compared with the control, but there was no difference (*P* > 0.05) among the AC, CN, and NRC100 treatments.

The mRNA levels for Cu transporter genes (Atox1 and Atp7a) were affected by treatment (Figure 2D). The mRNA levels for Atox1 and Atp7a were significantly up-regulated (*P* < 0.05) in AC, CN, and NRC100 treatments compared with the control, but there was no difference (*P* > 0.05) among the AC, CN, and NRC100 treatments.

### Meat Quality

There were no effects (*P* > 0.05) of different recommended amounts of Cu and Zn on carcass weight, backfat thickness, or LD muscle area in finishing pigs. Additionally, post-slaughter pH (45 min and 24 h), marbling, objective color measurements (L^*^, a^*^, and b^*^) of pork LD muscles, and shear force did not differ (*P* > 0.05) among the various recommended amounts of Cu and Zn (Table 8). Overall, pig carcass traits and meat quality were not affected by the recommended amounts of Cu and Zn during the finishing period.

**Table 8:** Effect of dietary Cu and Zn on the meat quality of finishing pigs (*n* = 8).

	**Dietary supplementation**					
**Items**	**Control^**1**^**	**AC^**2**^**	**CN^**3**^**	**NRC100^**4**^**	**SEM ±**	***P-*value**
Carcass weight, kg	75.74	76.62	76.45	76.89	3.272	0.137
Backfat thickness, mm	19.97	21.36	19.74	20.03	1.138	0.422
Loin muscle area, cm^2^	37.67	38.59	37.84	37.63	1.547	1.036
pH (45 min)	6.78	6.84	6.53	6.61	0.174	0.437
pH (24 h)	5.57	5.58	5.49	5.51	0.051	0.507
L*	52.66	55.37	54.29	54.67	0.693	0.448
a*	15.17	16.22	15.97	15.83	0.427	0.335
b*	5.24	5.65	5.14	5.12	0.627	0.657
Marbling	1.21	1.47	1.38	1.44	0.270	0.353
Shear force (*N*)	28.08	29.34	27.91	27.86	3.723	1.106

^a, b, c^>Means within a row without a common superscripted letter are significantly different (P <0.05). NCG-Cu, cupreous N-carbamylglutamate chelate; Zn-Met, zinc-methionine chelate. ^1^Control = basal diet (without added Cu or Zn). ^2^AC = basal diet + 35 mg of supplemental Cu/kg from NCG-Cu + 150 mg of supplemental Zn/kg from Zn-Met (actual levels used in Chinese commercial feed enterprises). ^3^CN = basal diet + 3.0 mg of supplemental Cu/kg from NCG-Cu + 43 mg of supplemental Zn/kg from Zn-Met (minimum recommended levels in Bulletin No. 1224 of the Ministry of Agriculture of the People's Republic of China). ^4^NRC100 = basal diet + 3.5 mg of supplemental Cu/kg from NCG-Cu + 50 mg of supplemental Zn/kg from Zn-Met (100% of NRC requirements for Cu and Zn). Meat acidity was measured with a pH meter (Matthaus pH Star, Germany) 45 min and 24 h after slaughter. Meat color traits (lightness L^*^, redness a^*^, and yellowness b^*^) were measured by a reflectance spectrophotometer Minolta CR-410 (Konica Minolta Sensing Inc., Osaka, Japan); marbling scores (ranging from 0 to 3, with 0 = absent and 3 = overly abundant) were subjectively evaluated; muscle shear force was determined with aGR-150 Warner-Bratzler Shear Machine (Shakopee, USA).

## Discussion

In China during recent years, Cu and Zn in excess of the MA-PRC recommendation levels have been added to pig diets to promote growth and reduce problems with diarrhea in commercial production (7). Numerous studies have shown increased growth and reduced diarrhea in young pigs fed diets supplemented with Cu and ZnO from either inorganic or organic sources (1, 6, 11, 18, 25), and some research has also indicated that dietary supplementation with various sources of Cu and Zn has no effect on growth performance in finishing pigs (10, 13), but the effects of the MA-PRC-recommended levels and the actual levels used by Chinese feed enterprises in feed for finishing pigs remain unclear. However, the results of the current study showed that dietary supplementation with different recommended levels of Cu and Zn had no effects on ADFI, ADG, or G/F in finishing pigs. The discrepancies may be because a typical corn-soybean meal diet fortified with limestone and dicalcium phosphate may have adequate innate Cu and Zn to support the growth of finishing pigs.

SOD, GSH-P*x*, MDA, GSH, T-AOC, and Cu/Zn SOD are the main antioxidant enzymes in mammals, and their activity levels are commonly used to assess body antioxidant status (25, 35). The present study showed that feeding the AC diet (35 mg/kg NCG-Cu + 150 mg/kg Zn-Met) brought a significant increase in the level of GSH-P*x* in the duodenum and tended to increase the level of Cu/Zn SOD in the duodenum compared with the other diets. A previous study showed that total liver GSH levels were lower in pigs fed diets supplemented with 225 mg/kg of CuSO_4_ than in pigs fed control diets, but total liver GSH did not differ between pigs fed the control diet and those supplemented with 225 mg/kg of TBCC (18), another study showed that T-AOC, Cu/Zn SOD, and GSH-P*x* activity levels in the serum and liver of weaned pigs receiving 100 mg/kg of chitosan-Zn chelate (CS-Zn) were higher than those of the pigs fed 100 mg/kg of ZnSO_4_,but weaned pigs fed 100 mg/kg of dietary CS-Zn also showed lower hepatic MDA levels than weaned pigs fed other diets (36). Cu/Zn-SOD could be used as a biological indicator to evaluate Cu and Zn status in the animal body, and the results also confirmed that the activity of Cu/Zn-SOD is affected by dietary Cu and Zn levels, as shown by many previous studies (18, 36, 37). These discrepancies in antioxidant capacity may be due to differences in the sources, age, dosage, or purity of the Cu and Zn used in the different experiments (1, 4). These results indicated that the antioxidant capacity of piglets, except for GSH-P*x*, was similar in pigs fed the Cu and Zn levels recommended by the NRC (2012) and the MA-PRC and the actual levels used by the Chinese feed industry.

Numerous studies have shown that excess consumption of minerals, such as Cu, Fe, Zn, or NCG-Cu, by pigs in different life stages can lead to liver mineral storage increase and thus can interact with and decrease the levels and function of other minerals (1, 4, 38–40). The present study clearly showed that the AC diet (35 mg/kg NCG-Cu+150 mg/kg Zn-Met) and CN diet (3.0 mg/kg NCG-Cu+43 mg/kg Zn-Met) produced a significant increase in the level of liver Zn, but the levels of Cu, Fe, and Mn did not differ among the four diets. Previous studies showed a large increase in liver Cu levels together with an increase in kidney Cu level in weanling pigs fed a high-concentration Cu diet (1, 16), which is not consist with the results of the present study. However, some studies also showed that liver Zn levels increased as dietary micromineral levels increased, as well as when Zn was specifically added to the diet using organic mineral sources at different mineral diets levels, but liver, kidney, and heart Cu and Mn concentrations were similar at the various micromineral levels in grower-finisher pigs (10, 13, 38), which is consistent with the results of the present studies. Meanwhile, in another study, liver Cu was decreased but kidney Cu was increased in response to a supplemented pig diet containing 15 mg/kg Cu and 27 mg/kg Zn with 100 mg/kg Zn (41). These discrepancies in organ mineral status may be due to differences in the sources, age, dosage, or purity of the Cu and Zn used in the different experiments, as well as a potential physiological response to protect against or prevent toxic effects when excessive levels of Cu, Fe, or Zn were fed in pigs (1, 4, 10). This risk should be studied further in relation to the reduction of Cu and Zn levels in pig diets.

Cu, Zn, and other mineral additives that are not absorbed by livestock are directly excreted in the feces when minerals are supplemented in excess of the animal's requirement, regardless of the use of inorganic or chelated sources (1, 4, 7). The present study clearly shows that reducing dietary Zn and Cu according to NRC100 (100% of NRC reference levels) or CN (MA-PRC reference levels) dietary standards is an effective method of reducing fecal excretion of Cu and Zn.Previous studies showed that Cu and Zn excretion could be reduced by at least 40% by reducing the added mineral levels in commercial feed (25 mg/kg of Cu and 150 mg/kg of Zn) to the levels recommended by the NRC (1998) (15). Numerous prior studies have confirmed that weaned pigs excrete decreased fecal Zn and Cu concentrations when supplemented with CuSO_4_ and NCG-Cu (1), as opposed to other mineral sources, and that reduced levels of Fe, Zn, and Cu are excreted in the feces of grower-finisher pigs when equivalent inclusion levels of organic Zn and Cu are fed (42). Therefore, reducing the Cu and Zn in pig feces is very important because the fecal metal content is not applied to crop land and is directly discharged into the environment, leading to soil pore blockage that reduces the air and water permeability of soil and polluting both surface water and groundwater (1, 7, 9). Additionally, evaluating and replacing the recommendations of different countries regarding levels or sources of Cu and Zn in the diet also reduce fecal excretion of minerals, such as Fe, Mn, and Cu (7, 10, 13). Therefore, in the future, the actual levels of Cu and Zn used by Chinese commercial feed enterprises should be reduced routinely, and other effective strategies to reduce micromineral excretion should be selected on the basis of realistic assumptions about environmental pollution.

A previous review article has shown that feed additives can affect digestive function and mucosal response in pigs at different life stages (43). Some studies have shown that Zn and Cu can affect variations in digestive enzyme activity in the pancreatic tissue of young pigs and rats, but lack of Cu on the enzyme activity in pancreatic homogenate (6, 16, 20, 44, 45), and some studies have shown that Zn and Cu can also affect variations in digestive enzyme activity in the intestinal contents of pigs and rats (6, 16, 45, 46). There was no effect of Zn on digestive enzyme activity in digesta samples, in contrast to data from a rat study showing that 1,000–5,000 mg/kg of dietary Zn can result in increased enzyme activity in pancreatic tissue as well as intestinal contents (6, 45). Excess amounts of Zn have been shown to decrease enzyme secretion in sheep (46). One study on weanling pigs has shown that addition of 15–250 mg/kg of Cu had no effect on trypsin, chymotrypsin, or amylase activity in the small intestine or on any of the five aforementioned enzymes in the pancreas but increased lipase and phospholipase A activity in the small intestine (16). In the current study, the levels of protein and mRNA expression of trypsin and APN exhibited significant up-regulation, but the levels of protein and mRNA expression of lactase, sucrase, maltase, and lipase did not differ in the duodenum intestinal mucosa. However, these data are not informative because the protein and mRNA expression levels of digestive enzyme were not measured in pancreatic secretion. Further studies are needed to elucidate the effect of different recommended levels of Zn and Cu on the synthesis and secretion of digestive enzymes from the porcine pancreas during different life stages.

Previous studies in rodent and pig models have shown that Zn homeostasis is affected by dietary Zn levels and by reduced absorption from the gut lumen (19, 26, 47); the uptake and tissue distribution of Zn rely mainly on two families of Zn transporters (48). The ZIP4 transporter is mainly involved in Zn uptake from the GIT, whereas ZnT1 can decrease intracellular Zn levels, and ZnT2 can decrease zinc levels in specific organelles (26, 48). The MT family is a family of cysteine-rich, low-molecular-weight proteins (~7 kDa), consisting of different isoforms and known to be involved in the regulation of Zn and Cu homeostasis and other metal transfer (48–50), but detection of MT by Western blotting is very difficult (51). Previous studies in rats have also shown that Zn uptake is transporter mediated at low to normal Zn levels but may also proceed by passive diffusion at higher Zn levels (52, 53). In the current study, the mRNA levels for ZnT1, ZnT2, ZIP4, and MT in the AC, CN, and NRC100 treatments were significantly up-regulated (*P* < 0.05) compared with the control (Figure 2C). The mRNA levels of intestinal ZnT1were up-regulated in the presence of a high zinc supply (5, 30, or 180 mg Zn/kg diet) and did not differ between the 30 and 180 mg Zn/kg groups but had no effect on liver ZnT1, consistent with previous studies in rodents (54). The expression of ZnT2 was down-regulated only by dietary Zn, which is not consistent with previous studies that mainly implicated it in Zn transport into intracellular vesicles (48). Some studies have shown that ZIP4 gene expression is down-regulated in IPEC-J2 cells (0~200 μmol/L Zn) and the pig small intestine (3,100 mg/kg dietary Zn) and under enterotoxigenic *Escherichia coli* (ETEC) K88 exposure (19, 55), which is not consistent with the current study. The gene expression level of ZIP4 is regulated by a series of Zn-dependent transcription factors (56), for example, *Krüppel-like factor 4* (KLF4), and will require further analysis in future studies. This is in concordance with recent findings showing that high levels of dietary or pharmacological Zn can increase MT mRNA levels in the tissue of weaned pigs and in theIPEC-J2 cell line (19, 57, 58). The increased MT expression levels after feeding high levels of dietary Zn to pigs may be related to enhanced growth (39, 50), but it remains unclear whether there is a direct relationship between MT and growth performance in pigs at different life stages, and further analyses will be required in future studies to clarify this point.

The Cu transporter and chaperone protein play such an important role in Cu acquisition, distribution, and utilization that this transporter has been used to evaluated the bioavailability of and nutritional need for Cu in pigs (26). Cu can enter the cell and can be delivered by ATP7a in intestine and ATP7b in liver through the chaperone protein Atox1 (59); the duodenum is the most important site of Cu absorption in rats (26). Previous work has shown that high levels of dietary Cu intake can decrease Cu absorption in humans (60), and numerous studies in cell models have shown that Cu uptake via Ctr1 is saturable (26, 61). In the current study, the duodenal mRNA levels for Atox1 and Atp7a were significantly up-regulated in the AC, CN, and NRC100 treatments compared with the control (Figure 2). Previous work from weanling pigs has shown that the duodenal mRNA levels of duodenal Atox1 were down-regulated in pigs receiving 225 mg/kg of CuSO_4_ or 225 mg/kg of TBCC compared with a control diet, but duodenal ATP7a did not differ among the three diets (25), which is not consistent with the current study regarding the duodenal mRNA levels of ATP7a and Atox1. In another study, the gene expression of Atp7a was measured in the livers of weanling pigs aged ~57 days, but the mRNA levels of Atp7a did not differ depending on the source or level of Cu (18), which is inconsistent with the results of the current study. This difference suggests that gene expression of Atp7a and Atox1 in liver and duodenal was affected by the age of pig and different tissue site (25, 26). These results showed that feeding different recommended levels of Cu and Zn resulted in modulation of duodenal Atox1 and ATP7a at the transcription level. Further work is needed to evaluate these transporters at the transcription level under similar dietary conditions with individual or combined Cu and Zn supplementation.

It has been widely demonstrated that the levels of micromineral are not related to meat quality or carcass characteristics (10, 62), and this lack of significant differences indicates that diets following the dietary micromineral recommendations of different countries should be studied further. In the current study, there was no effect of different recommended amounts of Cu or Zn on pig carcass traits or meat quality in finishing pigs (Table 8).

## Conclusions

Overall, these results indicate that diets supplemented with different recommended levels of Cu and Zn did not have a negative effect on growth performance or meat quality. However, the different recommended levels of Cu and Zn affected tissue mineral status, mineral excretion, digestive enzyme activity, and metal transporters in finishing pigs. These results indicated that the levels of supplemental Cu and Zn routinely used by Chinese commercial feed enterprises should be reduced in order to cut fecal excretion of Cu and Zn, although AC diets can increase antioxidant capacity, digestive enzyme activity, and metal transporter gene expression in finishing pigs. This research will help guide the MA-PRC recommendations for Cu and Zn supplementation in finishing pig diets and the actual levels used by Chinese commercial feed enterprises to better reflect practical conerns about environmental pollution.

## Data Availability Statement

The original contributions presented in the study are included in the article/supplementary material, further inquiries can be directed to the corresponding author/s

## Ethics Statement

This study was conducted in accordance with the guidelines of the Laboratory Animal Ethical Commission of the Chinese Academy of Sciences. All experimental animals used in this study were treated humanely followed the Animal Welfare Committee of the Institute of Subtropical Agriculture (ISACAS Animal Welfare Protocol #2016ISA0728), Chinese Academy of Sciences, Changsha, China.

## Author Contributions

PL and ML conceived and designed the experiments. PL performed the experiments, analyzed the data, and wrote the first draft of the manuscript. YL and WT contributed reagents, materials, and analytical tools. All authors contributed to the article and approved the submitted version.

## Funding

This research was supported by the Key projects of Guangxi Natural Science Foundation (2020GXNSFDA297002), Technological innovation plan of Changsha County Science and Technology Bureau (2021055), Fujian Provincial Science and Technology Department and Chinese Academy of Sciences Supporting Project of STS Program (2021T3051), local science and technology development fund project of the Central Committee of Guangxi Zhuang Autonomous Region (Gui ke ZY21195054), Application and industrialization of scientific and technological achievements in Guizhou Province (Qian ke he cheng guo [2021] yi ban 037), the Joints Funds of the National Science Foundation of China (Grant No: U20A2054), the National Natural Science Foundation of China (31960666), Science and education joint project of Hunan Natural Science Foundation (2020JJ7047), Scientific research project of Hunan Provincial Department of Education (19C1102).

## Conflict of Interest

WT was employed by company Hunan Tianxin Seed Industry Co., Ltd. The remaining authors declare that the research was conducted in the absence of any commercial or financial relationships that could be construed as a potential conflict of interest

## Publisher's Note

All claims expressed in this article are solely those of the authors and do not necessarily represent those of their affiliated organizations, or those of the publisher, the editors and the reviewers. Any product that may be evaluated in this article, or claim that may be made by its manufacturer, is not guaranteed or endorsed by the publisher.

## Abbreviations

Cu: copper
Zn: zinc
BW: body weight
ADG: average daily gain
ADFI: average daily feed intake
F/G: feed-to-gain ratio
LD: longissimus dorsi
ICP-OES: inductively coupled plasma optical emission spectrometry
AA: amino acid
GIT: gastrointestinal tract
NCG-Cu: cupreous N-carbamylglutamate chelate
Zn-Met: zinc-methionine chelate
MA-PRC: Ministry of Agriculture of the People's Republic of China
NRC: National Research Council
PBS: phosphate buffer saline
SOD: superoxide dismutase
GSH-P*x*: glutathione peroxidase
MDA: malondialdehyde
GSH: glutathione
T-AOC: total antioxidant capacity
Cu/Zn SOD: Cu/Zn superoxide dismutase
APN: aminopeptidase N
ZnT1: zinc transporter SLC30A1
ZnT2: zinc transporter SLC30A2
ZnT5: zinc transporter SLC30A5
ZIP4: zinc transporter SLC39A4
DMT 1: divalent metal transporter 1
MT: metallothionein 1
Ctr1: Cu transporter 1
Atox1: antioxidant 1
Atp7a: Cu^2+^-transporting α-polypeptide ATPase
Atp7b: Cu^2+^-transporting β-polypeptide ATPase
Cox17: cytochrome c oxidase assembly protein 17
TBP1: TATA box binding protein
RPL4: ribosomal protein L4
Hprt1: hypoxanthine phosphoribosyltransferase 1
β-actin: beta-actin
L^*^: lightness
a^*^: redness
b^*^: yellowness
ANOVA: analysis of variance
CS-Zn: chitosan-Zn chelate
TBCC: tribasic Cu chloride
ETEC: enterotoxigenic *Escherichia coli*
KLF4: Krüppel-like factor 4.
